# Curated cauldrons: Preserved proteins from early copper-alloy vessels illuminate feasting practices in the Caucasian steppe

**DOI:** 10.1016/j.isci.2023.107482

**Published:** 2023-08-24

**Authors:** Shevan Wilkin, Peter Hommel, Alicia Ventresca Miller, Nicole Boivin, Antonella Pedergnana, Natalia Shishlina, Viktor Trifonov

**Affiliations:** 1 Institute of Evolutionary Medicine, University of Zurich, Zurich, Switzerland; 2 Max Planck Institute of Geoanthropology, Jena, Germany; 3 Australian Research Centre for Human Evolution (ARCHE), Griffith University, Brisbane, QLD, Australia; 4 Department of Archaeology, Classics and Egyptology, University of Liverpool, Liverpool, UK; 5 Department of Anthropology, University of Michigan, Ann Arbor, MI, USA; 6 Museum of Anthropological Archaeology, University of Michigan, Ann Arbor, MI, USA; 7 Department of Anthropology, National Museum of Natural History, Smithsonian Institution, Washington, DC, USA; 8 School of Social Science, University of Queensland, Brisbane, QLD, Australia; 9 Griffith Sciences, Griffith University, Brisbane, QLD, Australia; 10 State Historical Museum, Moscow, Russia; 11 Peter the Great Museum of Anthropology and Ethnography (the Kunstkamera), St Petersburg, Russia; 12 Institute for the History of Material Culture, St Petersburg, Russia

**Keywords:** Paleobiochemistry, Archeology

## Abstract

Large metal and metal-alloy cauldrons first appear on the far western steppe and Caucasus region during the Maykop period (3700–2900 BCE); however, the types of foods or beverages cooked in and served from these vessels have remained mysterious. Here, we present proteomic analysis of nine residues from copper-alloy cauldrons from Maykop burial contexts where we identify muscle, blood, and milk proteins specific to domesticated, and possibly wild, ruminants. This study clearly demonstrates that the earliest, large-volume feasting vessels contained both primary and secondary animal products, likely prepared in the form of a stew.

## Highlights

• Copper-alloy vessels provide extended preservation of organic residues
• Muscle, blood, and milk, likely from a cooked soup, found in 5000-year-old cauldron
• Blood and milk from ruminants, likely domesticates, were included in the residue

## Graphical abstract

**Figure:**
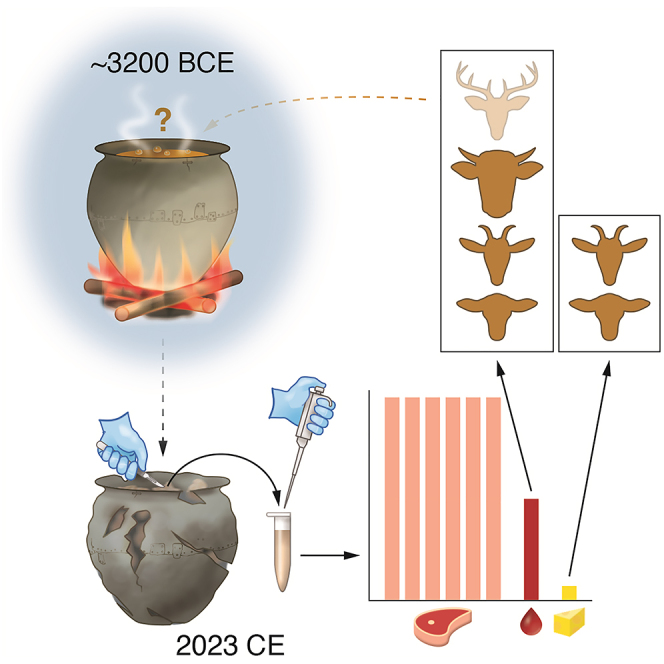

## Introduction

Large metal vessels, kettles, kazans, or cauldrons, are found in later prehistoric societies across Northern Eurasia and hold a particular fascination for students of the past.^1^ These often-ostentatious containers are at once familiar and enigmatic, connoting instability, mixing and magic, creation, destruction, and rebirth.^2^^,^^3^ Though some of these associations are relatively recent, others have deeper roots, and the significance of these vessels for the societies that made them is evident from the outset. They would have been prized not only for their material value but also for the skills deployed in their production. Crafting a substantial hollow form out of metal is a technical feat that requires complex thin-walled casting, extensive cold-working, or some combination of both.^4^ Unsurprisingly, such vessels have been consistently associated with communal events where they could serve as visible symbols of power, highlighting differences in status among participants and reinforcing the social order.^3^^,^^5^^,^^6^

**Figure 1:**
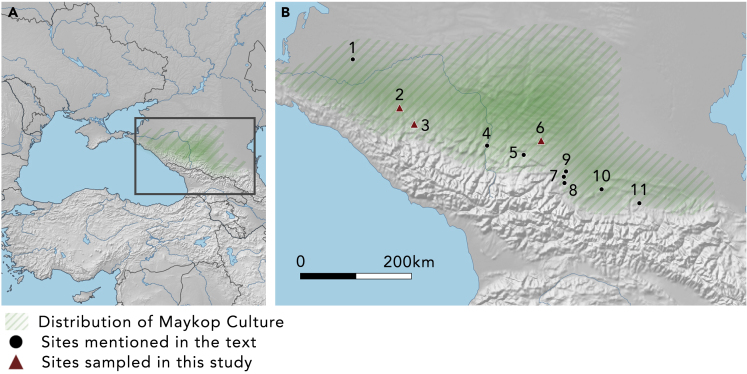
Location of study (A) Region of study on larger map. (B) Map of the Caucasus showing sites mentioned in the text, the primary distribution of the Maykop finds and sites where metal vessels have been recovered: 1–Staromyshastovskaya Hoard; 2–Maykop Kurgan (Oshad); 3–Klady Cemetery (including Tsarskaya); 4–Kubina; 5–‘Avtozapravki’, Kislovodsk; 6–Inozemtsevo; 7–Chegem IV; 8–Nalchik; 9–Kishpek; 10–Zamankul; 11–Bamut.

However, while their probable connection with rituals and/or feasting is widely accepted, the precise details of their function are essentially unknown, and whether they were used in the making and sharing of food, the preparation of intoxicants, or some other purpose remains debatable.^7^^,^^8^ The aim of this study was to attempt to resolve this uncertainty in the case of the earliest known metal cauldrons in Europe, associated with the funerary mounds of the Maykop culture (ca. 3700–2900 BCE) in the northern piedmont of the Greater Caucasus Mountains and the southern edge of the Eurasian steppe (Figure 1).

### Trends in the use and reuse of early metal vessels

Direct investigation of the roles of metal vessels in prehistoric society is complicated by a number of factors, not least that of survival. With the exception of gold and silver, which preserve well in most conditions, other metal artifacts are susceptible to corrosion and post-depositional damage;^9^ thin-walled objects—having both a large surface area and a small cross-sectional area—tend to be the most vulnerable. In addition, because of their significance to the societies that made them, they were often preserved as objects of value, curated as heirlooms, or deployed as material resources for trade and exchange.^10^ The idea of a prolonged use-life is reinforced by evidence of extensive repair seen on many vessels, suggesting that special care was taken to keep them in service.^1^ Circulating among the living, these objects may have endured beyond the lives of their owners, and even when irreparably damaged could have been recast into new forms.^11^ The decision to remove them from this cycle (e.g., to bury them with the dead) would not have been taken lightly, and as the “recovery” of metal items from earlier graves was common in prehistory, they may not always have stayed buried for long.^12^ All this may explain why relatively few of these artifacts have entered the archaeological record and, with limited evidence available, the precise origins of the first metal vessel traditions are likely to remain unclear. However, we can trace some general trends.

Although isolated finds of metal vessels are known from Anatolia and Mesopotamia around the turn of the third millennium BC,^7^^,^^13^ according to Reeves’^14^ comprehensive study, the *regular* appearance of metal vessels in Western Asia begins in the Anatolian Early Bronze Age II (i.e., after 2700 BC). However, the refinement seen in these early vessels suggests that traditions of production were already well established—an idea supported by widespread skeuomorphic representations of metal vessels in ceramic forms in the preceding centuries.^7^^,^^14^^,^^15^ To date, there is only one substantial corpus of metal vessels from the 4th millennium BCE—recovered from the burial mounds of the Maykop culture in the northern Caucasus^16^ (Figure 1).

### Metal vessels in Maykop society

**Figure 2:**
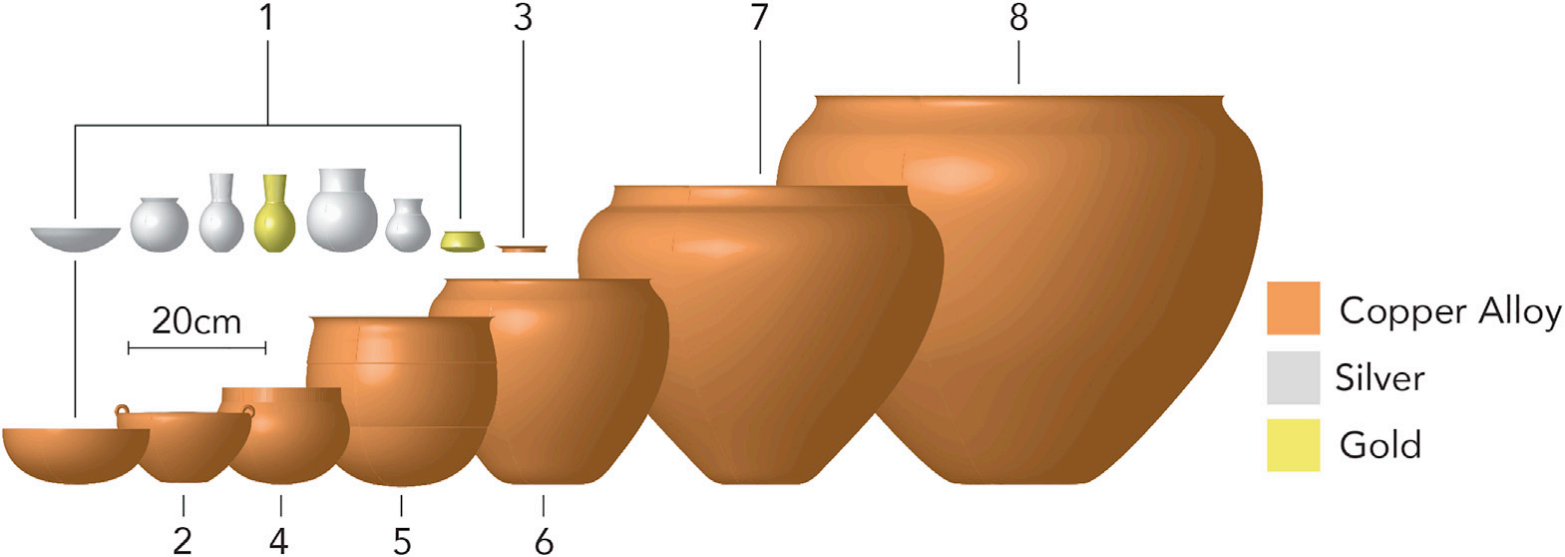
Showing a range of Maykop metal vessel forms (reconstructed after Korenevskiy 1988; 2003; Piotrovskij 2020; Rezepkin 2012): 1–Maykop Kurgan (Oshad); 2- Klady, k. 31, gr.5; 3- Klady, k. 11, gr. 5; 4–Zamankul, k. 1, gr. 70; 5–Inozemtsevo; 6–Bamut, k. 14; 7–Kishpek 2, k. 1, gr. 1; 8–Nalchik cist

Since the late 19th century, excavations along the northern piedmont of the Greater Caucasus range and the southern fringes of the Eurasian steppe have identified the remains of about 40 metal vessels in the burials and hoards of the so-called Maykop culture.^8^ These Maykop vessels are varied (Figure 2), both in their forms and their technological characteristics and from the moment of their discovery, their origins and significance have remained a focus of archaeological attention.^4^ The best known and most widely published are small cups and flasks of precious metal (silver and gold).^17^^,^^18^ These vessels, some of which were elaborately decorated with images of wild and domesticated animals, have been linked to early Mesopotamian and Anatolian assemblages which are typically dominated by cups and flasks and associated with drinking or the serving of liquids.^14^^,^^18^^,^^19^

**Figure 3:**
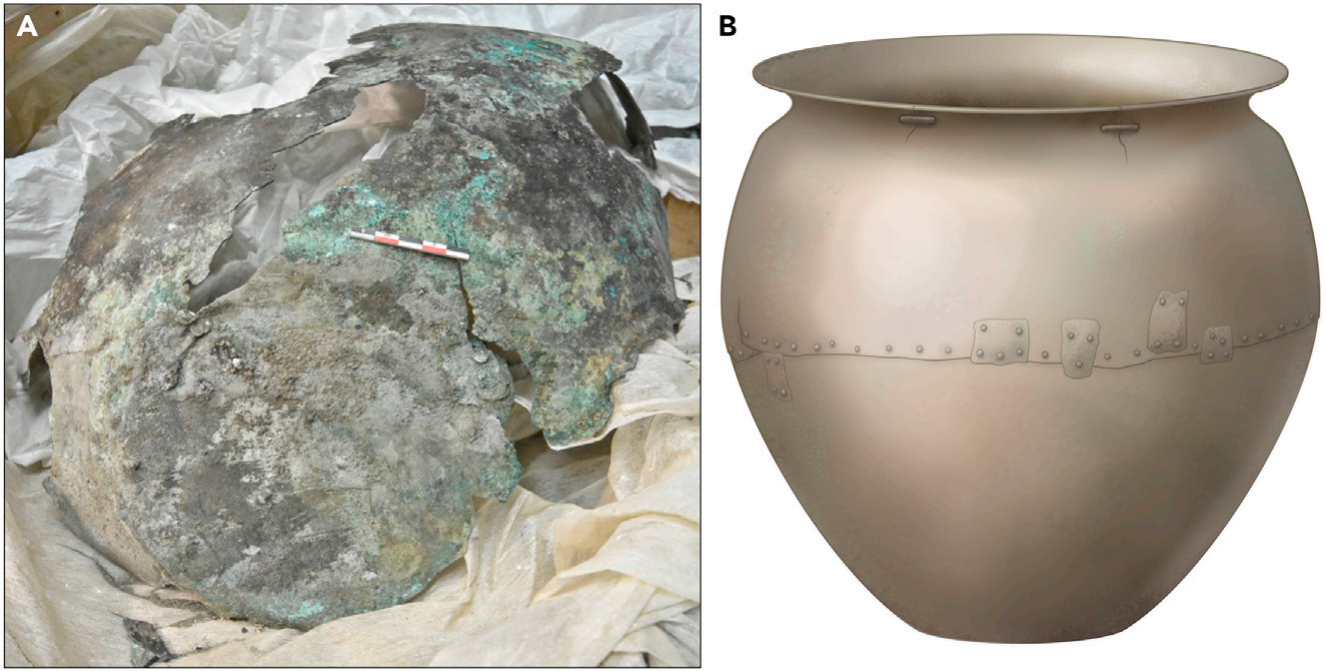
Copper-alloy vessel from Inozemtsevo (A) Photograph of the cauldron and what is left today. (B) Artistic reconstruction of the cauldron as it would have looked when in use.

Less well known, but equally remarkable are the large copper-alloy vessels, referred to as “cauldrons” found at the Maykop burial mound itself and a number of other sites in the region.^8^ The socio-symbolic importance of these vessels in contemporary society has been argued on the basis of their funerary context in association with burials of the highest elite^8^ and the extensive repairs they received during their lives. One of these cauldrons from the later Maykop burial at Inozemtsevo (analyzed in this study), for example, has several patches riveted along the main seam in the middle of the vessel, as well as at least two clips to hold together cracks developing at the rim (Figure 3). These large vessels form the focus of our study.

Though clearly significant to the people who made and used them, the practical function of these vessels remains essentially a matter of assumption, with generic references to the preparation of food and/or drink, supported with wider contextual references to “table sets” or the presence of other serving implements within the graves.^20^^,^^21^ Some vessels do show outward indications of use—indeed, as part of this study, we were able to directly date traces of soot commingled with corrosion products on the surface of the “cauldron” from the Maykop mound itself—however, while some role in heating seems plausible, this does not greatly increase our understanding of the *specific* functions of these vessels and only emphasizes the need for further research.

### Protein analysis in the study of ancient diets and metal vessels

Over the past decade, protein analysis has been applied to numerous archaeological materials, such as: human dental calculus,^22^^,^^23^^,^^24^^,^^25^^,^^26^^,^^27^ skeletal remains,^28^^,^^29^ mummified tissues,^30^ leather,^31^ parchment papers,^32^ and more. Dietary proteins have been of special interest, as they provide proxy information on subsistence strategies, for example, evidence for dairy consumption or grain use can imply cultivation practices and human-animal interactions, lending insights into domestication and the use of primary and secondary products. However, while these interests have increased our understanding of the survival of dietary proteins within certain contexts, the applicability of protein analysis on the interior surface residues of metallic vessels has yet to be thoroughly explored.

Copper is well known to preserve organic materials (e.g., leather and textiles) in close contact with it due to its naturally antimicrobial properties^33^^,^^34^^,^^35^^,^^36^^,^^37^ and it would be reasonable to expect that the same preservation would extend to other organic materials. Organic residues (fats and waxes) from cauldrons from the Late Bronze Age and subsequent periods (after 1500 BCE) have been successfully studied by looking at preserved fatty acid chains through gas chromatography-mass spectrometry which have reportedly identified materials such as, but not limited to: beeswax and tree resins,^38^^,^^39^ wine and/or vinegar,^40^ animal fats,^41^ and plant foods or beverages.^42^ These lipid-based methods are helpful in narrowing down elements within the residues, but do not generally provide the taxonomic resolution of DNA or protein analyses, which can potentially offer species and tissue-specific identifications.

The recovery of preserved proteins in archaeological samples is extremely variable and strongly dependent on the age of the sample, type of material, and the environmental conditions of the region. For example, while researchers are still actively exploring the exact nature of biomolecular preservation in archaeological materials, it has been shown that proteins from materials in cold environments fare better than those from consistently warm or hot climates. Furthermore, proteins contained within dental calculus, dental enamel, or ceramic residues with a calcified coating are held within a relatively closed system, and biomolecular materials (proteins, aDNA, metabolites) retain a higher level of preservation than those openly exposed to the elements.^26^^,^^43^^,^^44^^,^^45^

The potential for protein residue analysis in the study of ancient metal vessels has recently been demonstrated in a study by Carvalho et al.^37^ who successfully extracted and identified proteins from the corroded surface of a metal vessel recovered from a tavern in the Roman city of Pompeii (ca. 79 BCE). However, it is not clear how widely applicable this technique would be, or how far into the past it would remain effective. Our study provides an opportunity to explore the functional limits of this potentially game-changing methodological approach by examining some of the earliest surviving metal vessels in the European archaeological record to better understand their role in early steppe societies.

### Eating or drinking: Expectations and limitations

#### Eating

It is often assumed that large vessels were used for the processing, cooking, and/or serving of food for consumption. In this case, a broad spectrum of possible patterns might emerge from within any protein residues recovered from these vessels. However, as Eneolithic and Early Bronze Age societies in the western steppe and Caucasus were heavily reliant on proteins derived from animal tissues, such as meat, blood, and milk,^22^^,^^46^ it would be reasonable to expect animal proteins to be present in any residue related to food.

Although much is still not understood about which species were consumed, whether at everyday meals or feasting events, faunal remains at Maykop sites tend to be dominated by cattle and sheep/goat with small percentages of pig, and wild species, principally deer and/or tarpan (*Equus ferus*), all of which were possible food sources.^47^^,^^48^^,^^49^ Aside from the meat and blood of these species, secondary products of ruminants were commonly exploited in neighboring areas of Europe,^22^^,^^46^ and these vessels may have played an important role in the preparation and consumption of milk or dairy products—whether alone or added as a component of Maykop dishes.

Unfortunately, muscle proteins are extremely conserved between species, often only specific to the megaorder, and often match to 90% of vertebrates. This makes the identification of archaeological muscle proteins to a particular genus or species challenging, if not impossible. Conversely, milk and blood protein sequences are more variable, and offer a greater taxonomic resolution in the recovered peptides.

#### Drinking

If past vessels were used in the preparation or serving of alcohol or other intoxicants, the evidence might well be more elusive, but there would be the potential to recover residues dominated by plant or fermented milk proteins, and perhaps the enzymes or microbial proteins from bacteria and yeasts used to jump start fermentation. The provisional identification of cereal starch grains (see Trifonov^50^) in association with “drinking straws” from the Maykop kurgan itself adds another dimension to the possible range of vessel uses. Evidence of widespread plant cultivation within Maykop society is limited, but cereal grains (principally *Triticum aestivum* and *Hordeum vulgare)* have been recovered from contemporaneous sites on both sides of the Greater Caucasus^51^^,^^52^ and found in direct association with Maykop ceramics at the Sereginskoe settlement.^47^

In addition to plant-based beverages, milk was widely consumed in steppe populations by the Early Bronze Age, and was likely featured in Maykop dietary traditions. While milk can be consumed either fresh or as a diverse range of commonly consumed dairy products (yoghurt, cheese, kefir), it can also be turned into an alcoholic beverage. The sugars in milks are easily converted into alcohol through fermentation, which could have featured at feasts. If this was the case, we would expect to see milk whey proteins, which would allow us to determine which species the milks derived from.

Here, we present the earliest findings of dietary protein residues recovered from Eneolithic/Early Bronze Age metal vessels and consider their significance for our understanding of Maykop feasting practices.

#### Samples, dates, methods, and analysis

In order to better understand what was processed, cooked in, or served from these vessels, we conducted proteomic extractions and analysis of nine residue samples from the interior surface of seven copper-alloy “cauldrons” recovered from Maykop burials (Table 1): *Klady (Novosvobodnaya*) *Cemetery (including the Tsarskaya Kurgan*)—three residue samples from two large vessels recovered from Kurgan 11, Gr. 26 and Kurgan 31, Gr. 5 and three residue samples fragments from three unreconstructed vessels from Tsarskaya (Kurgan 1); *Maykop kurgan (Oshad)*—one residue sample and one sample of soot from the external surface of the same vessel (for dating); *Inozemtsevo Kurgan*—two residue samples taken from one cauldron (Figure 3) from the central burial.

**Table 1:** List of all samples, site, curation location, excavation date, laboratory ID, accession/archaeology ID, and sample weight in milligrams

Sample type	Archaeological Context	Curation Location	Date excavated	Lab number	Accession/Arch ID	Weight mg
14C Dating	Maykop kurgan, Primary burial	Hermitage	1897	P49393	M-1897/11/I	159
Cauldron residue	Maykop kurgan, Primary burial	Hermitage	1897	DA710	M-1897/11/I	10.5
	Inozemtsevo, Central Burial	SHM	1976	DA370	DA-INO0719-002.A	17
	Inozemtsevo, Central Burial	SHM	1976	DA377	DA-INO0719-003.A	10.1
	Tsarskaya, k. 1, Megalithic tomb (Klady Cemetery)	Hermitage	1898	DA707	TS-98/89/7	15.3
	Tsarskaya, k. 1, Megalithic tomb (Klady Cemetery)	Hermitage	1898	DA708	TS-98/89/4	21.5
	Tsarskaya, k. 1, Megalithic tomb (Klady Cemetery)	Hermitage	1898	DA709	TS-98/89/2	11.5
	Klady Cemetery (Kurgan 11, Gr. 26)	Hermitage	1898	DA711	Klady, k. 11	23.4
	Klady Cemetery (Kurgan 31, Gr. 5)	Hermitage	1979	DA712	Klady, k. 31.4	20
	Klady Cemetery (Kurgan 31, Gr. 5)	Hermitage	1979	DA713	Klady k. 31.3	8.8
Positive control	NA	NA	NA	DA715	NA	2.9
Extraction blank	NA	NA	NA	DA716	NA	NA

With the exception of the vessel from Kurgan 11 at Klady, which had been conserved in bandages coated with polyvinyl butyral, the analyzed samples were selected from vessels which had seen minimal curatorial treatment or handling. These were considered least likely to have been contaminated after excavation. Protein samples were collected from the interior surfaces of the vessels in sterile 2 mL Eppendorf tubes and shipped to the Max Planck Institute for the Science of Human History, Department of Archaeology, where proteins were extracted in a clean lab designated only for ancient proteomic analysis following an established protocol optimized for archaeological materials.^53^ The dating sample from the Maykop cauldron was prepared for analysis at the Oxford Radiocarbon Accelerator Unit at the University of Oxford using the “ZR” pretreatment method^54^ and rinsed three times with sodium hydroxide solution during the base step to remove excess base-soluble material.

## Results

**Figure 4:**
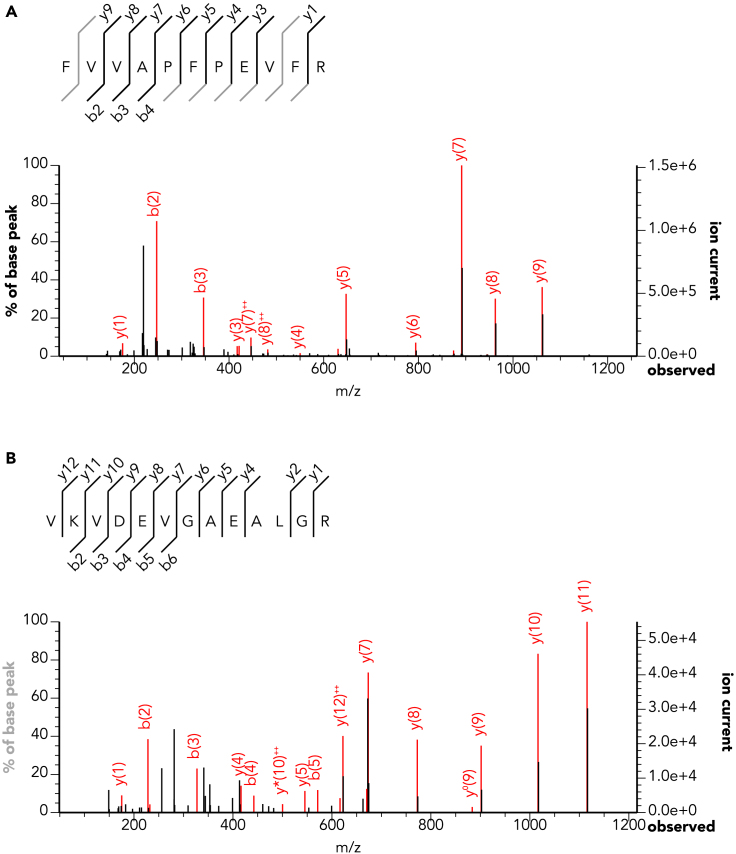
MS/MS spectra for peptides from the Inozemtsevo cauldron (DA 370) (A) Peptide from hemoglobin subunit beta-1, specific to Caprinae (sheep or goat). (B) Peptide from alpha-S1-casein, specific to Caprinae (sheep, goat). See supplement for additional spectra.

**Figure 5:**
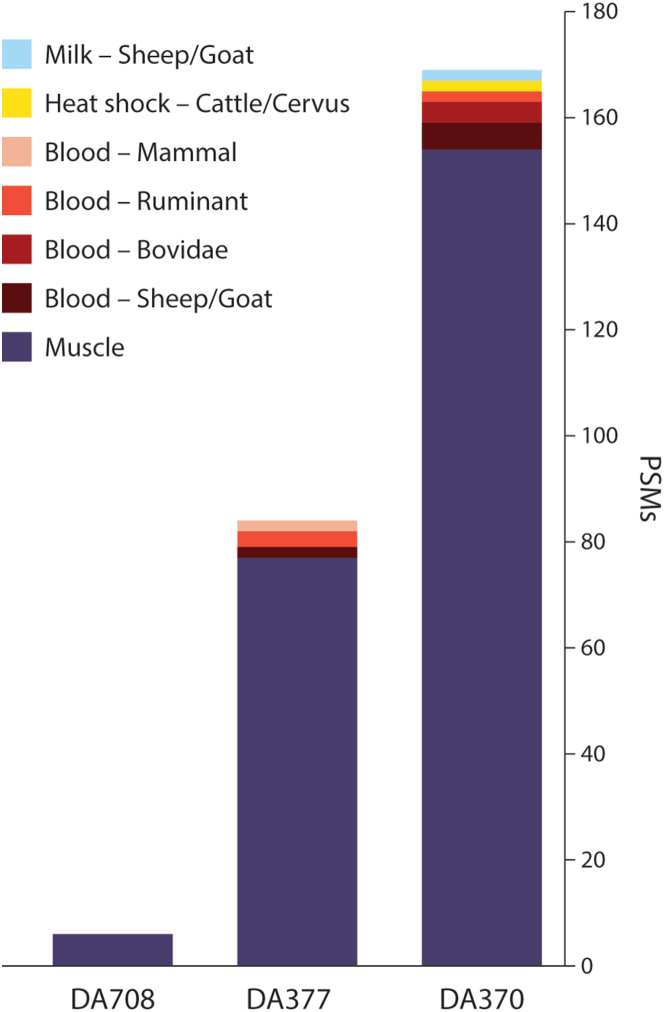
Bar chart showing the number of peptide spectral matches (PSMs) per sample for each tissue

Three of the eight samples analyzed produced identifiable dietary proteins. Of these, two were taken from the residues of one cauldron from Inozemtsevo, and the third sample was from a cauldron residue recovered from Tsarskaya (Table 2; Figures 4 and 5) All other samples only contained proteins from laboratory (trypsin, bovine serum albumin), environmental (soil microbiome bacteria), or handling contamination (collagens, keratins, etc).

**Table 2:** Recovered protein evidence from each sample

Site	Lab ID	Dietary Proteins	Species and tissue
Maykop kurgan	DA710	No	ND
Inozemtsevo	DA370	Yes	Muscle: Vertebrate; Blood: Pecora, Bovidae, Caprinae; Milk: Caprinae
Inozemtsevo	DA377	Yes	Muscle: Vertebrate; Blood: Pecora, Bovidae, Caprinae
Tsarskaya, k.1 (Klady Cemetery)	DA707	No	ND
Tsarskaya, k.1 (Klady Cemetery)	DA708	Yes	Muscle: Vertebrate
Tsarskaya, k.1 (Klady Cemetery)	DA709	No	ND
Klady Cemetery (Kurgan 11)	DA711	No	ND
Klady Cemetery (Kurgan 31)	DA712	No	ND
Klady Cemetery (Kurgan 31)	DA713	No	ND

For full data on each recovered protein and peptide spectral match, see Table S1.

The two residues from the Inozemtsevo cauldron were both recovered from the inside surface of the cauldron. A range of animal muscle proteins were recovered (tropomyosin beta chain, tropomyosin alpha-1 chain, tropomyosin alpha-3 chain, myosin-7, myosin-6, myosin-1, myosin light chain 3) but their protein sequences are conserved (shared) by most vertebrate species. One protein found in multiple animal tissues (muscle, many organs, skin), heat shock protein beta-1, was also recovered with an amino acid sequence specific to either the subfamily Bovinae (cow, yak, water buffalo) or the genus Cervus (deer).

Three different blood proteins were also identified: hemoglobin subunit alpha-1, specific to the Bovidae family (cattle, sheep, and goat); hemoglobin subunit beta, slightly less taxonomically specific peptide sequences matching to all even-toed ruminants (Pecora); and serum albumin found to be specific to the subfamily Caprinae, indicating that it derived from either a sheep or goat. Serum albumin from either cattle or humans are considered laboratory contaminants and are routinely excluded from results; however, as our peptide identifications were specific to either sheep or goat, they are not considered to be laboratory contamination and we have included them in our results. Three peptides from the milk protein alpha-S1-casein were recovered, and these are specific to Caprinae subfamily, indicating that milk from either sheep or goat was present.

The third sample, from a residue from the Tsarskaya burial mound in the Klady cemetery, was also taken from the interior of the cauldron. This residue contained just two different muscle proteins, tropomyosin alpha chain and tropomyosin beta chain, similar to the samples from Inozemtsevo. However, as these are very conserved sequences, shared between most vertebrates, they cannot be identified to any specific family, genus, or species.

The dating sample (P49393), taken from the outside of the same cauldron as sample DA710 from the Maykop Kurgan (Oshad), yielded a radiocarbon date of 4645 ± 23 bp (OxA-X-3106-13): 3520–3350 calBCE. This date correlates well with the anticipated (archaeological) date for the burial and is the first direct date for this important burial complex. The date was given an advisory OxA-X prefix by the laboratory, having produced a low carbon yield (%C = 8.9%) relative to sample pre-testing due to exogenous low carbon material in the substrate (likely corrosion products from the cauldron adhering to the soot layer); however, the carbon yield is not low enough to place doubt upon the recovered date.

## Discussion

### Recovered proteins mirror archaeological data

Protein results from the Maykop cauldron residues fit solidly within existing evidence of archaeofaunal remains from the Eneolithic sites in the northern Caucasus region and Maykop sites in the late Eneolithic/EBA. The notion of large vessels for the cooking and serving of meat and other animal products, as part of communal ceremonies or feasts, fits well with traditional archaeological expectations and it seems within reason to situate these feasts within the social context of funerary rituals. Animal bones are often found deposited within Maykop burials, though they are not always reliably identifiable. The recovered taxonomic identifications to Pecora (all even-toed ruminants, including deer and elk), Bovidae (cow, sheep, or goat), Bovinae (cow, yak, water buffalo), and Caprinae (sheep or goat) also dovetail with the species identified in recently published proteomics studies of human dental calculus recovered from individuals from both the Caucasus and broader Bronze Age Pontic-Caspian steppe.^22^^,^^46^ However, while most dietary proteins identified from dental calculus derive from ruminant milk, within these cauldrons the evidence is indicative of meat, providing insights into the specific uses of these vessels and further illuminating the range of culinary diversity. The findings of the Inozemtsevo cauldron are particularly interesting, as the recovered evidence contained proteins from caprines (sheep or goat) and either bovids (most likely cow) or cervids, which could represent the cooking of both wild and domesticated fauna. While we cannot determine whether milk and meat from multiple species were cooked simultaneously, or if they represent a palimpsest of distinct cooking events, it is clear that the users exploited multiple animal species and tissues for dietary resources.

**Figure 6:**
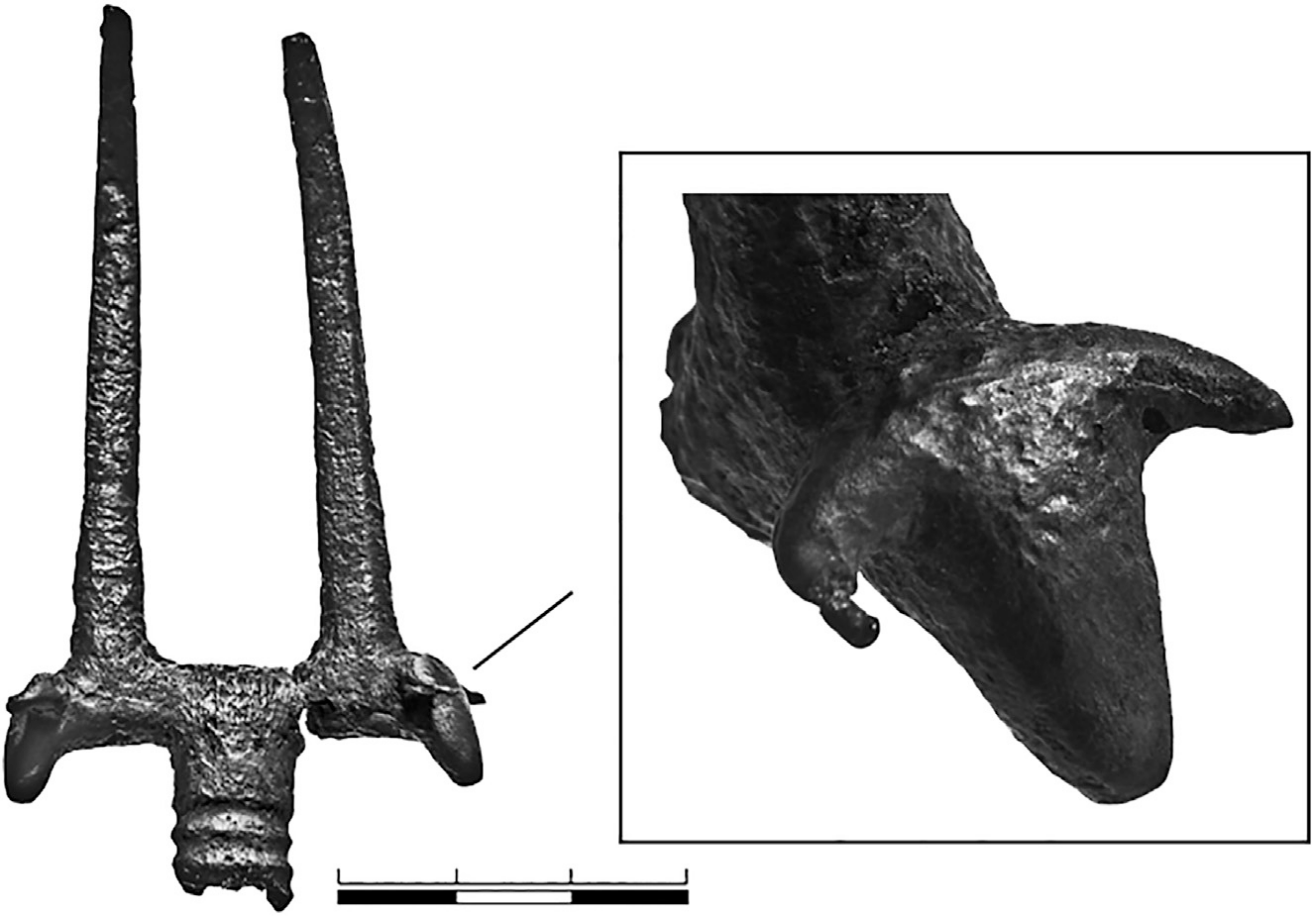
A two-pronged fork with bovine protomes from Kurgan 28, Klady cemetery (Photo: V. Trifonov^60^

Although consistent with the zooarchaeological record from Maykop sites, this variety is at odds with the decidedly bovine focus of elite material culture. Carefully crafted figurines of bulls in gold, silver, and stone serve to emphasize the importance of cattle seen in other aspects of funerary practice, including the incorporation of bucrania and/or nose rings into burials across the Maykop area.^55^^,^^56^ The plausible connection between cattle and cauldrons is perhaps most clearly expressed in the pronged forks or horn-like hooks found within Maykop burials, often in association with large metal vessels. It has long been suggested that these artifacts may have been used to manipulate lumps of hot meat during cooking (e.g., Kuftin 1949, 280–281^57^). At Tsarskaya, for example, three such objects were found together with three cauldrons, one for each vessel.^20^ Similar objects have been found at Inozemtsevo and an example from Kurgan 28 in the Klady cemetery (Figure 6) actually has bovine protomes (a human or animal head/torso) at the base of the tines.^58^^,^^59^ With all this bovine imagery in mind, the rather more caprine aspect of the residues from Inozemtsevo is intriguing.

It is possible that this represents an underrepresentation of bovine-specific proteins in our results. BSA is a widespread contaminant in proteomics facilities, and we cannot reasonably determine recovered BSA peptides in our samples derived from consumed fauna or laboratory contamination. The same issue does not affect serum albumin proteins specific to Caprines, which is not widely used in laboratories; these sheep/goat blood protein identifications are reliable as coming from animal tissues used in food preparation. However, other proteomic evidence for the consumption of cattle (cow, yak, or buffalo) in these vessels was very limited. Indeed, heat shock protein beta-1, found in the residues from Inozemtsevo, matches both to Bovinae (cattle) and predicted sequences for Cervus (deer). This, combined with two additional proteins that match to either Caprinae or Cervus, may equally reflect the consumption of wild, hunted animals. It is important to note that these Cervus sequence matches from the database have been translated from genomes, but not yet confirmed with mass spectral data. While it would be tempting to prefer the interpretation of cattle on the basis of the wider archaeological context, the presence of wild animals, deer or elk in the samples is also entirely possible. There is ample evidence for deer exploitation, for food and for secondary products (e.g., antlers) in Maykop society. At Tsarskaya, for example, red deer and sheep remains were placed at the entrance of the dolmen and interpreted as an offering to the dead.^59^ While no offerings of deer were identified at Inozemtseva, antler hammers (potentially for stone working) were placed in the main burial, and various deposits of wild and domesticated animal bone, including sheep and wild horse (*Equus ferus*), were found elsewhere in the mound.^58^^,^^59^ While it remains unclear how all of these animals fitted into the contemporaneous cookery, the potential to uncover not only the types of food that were prepared and consumed from these vessels but also details of culinary practices is enticing.

The well-used and repaired condition of these vessels also tells a compelling story. The long-term reuse of this vessel confirms its significance, reflecting not only their material value but also the significant skill required to produce these vessels.^4^ Although the focus of this study was on larger vessels, the range of metal containers employed within Maykop society was much greater, not only cauldrons but also bowls, cups, and serving platters (Figure 2). Further research into the varied role of vessels in Maykop society will deepen our understanding of contemporary food preparation and dining practices.

### Protein preservation prolonged through exposure to metal alloys

The naturally antibacterial environment provided by the copper-alloy of the cauldron acted as a preservation agent for the proteins contained within thousands of years ago. A previous study into proteins recovered from ancient ceramics, such as at Çatal Höyök, provided similar results, with the recovery of animal meat and milk proteins. While proteins do not regularly preserve on the surface of buried ceramics, proteins recovered from Çatal Höyök vessels were encased in calcified deposits that formed over the life of the bowls.^61^ Unique situations, such as the discovery of residues in corroding metal vessels or calcified residues on ceramics, provide a snapshot of ancient cooking and food preparation practices that would otherwise be unavailable.

Similar to the Çatal Höyök study,^43^ we found that burned or charred residues from the Maykop and Tsarskaya vessels contained very few if any recoverable proteins. Proteinaceous materials are often not preserved after the burning process,^62^ often leaving sooty residues without recoverable proteins. In our samples, most of those that were burned did not contain any indication of dietary proteins and overall protein recovery was limited to laboratory and environmental contamination, and the single burned sample that did yield ancient proteins, there were far fewer recovered than in the unburned residues.

### Conclusion

Meat, blood, and milk proteins indicate that these specific Maykop cauldrons were utilized for cooking or serving primary and secondary products of ruminants, likely a form of stew. While the small number of residues sampled in this study indicate that these vessels were used for preparation or serving of a meat-based dish, it remains possible that other vessels could have been used to prepare other types of food or beverages.

Biomolecular studies of ancient food and beverage vessels used in preparation, storage, or serving can increase our understanding of the individual foodways used by early populations. The preservation allowed by copper and other metal vessels opens up new avenues of study for ancient food preparation, processing, and serving. Future studies covering a wider range of Maykop vessel forms may provide additional insights into the practices and preferences of cooking and consumption on the early steppe and beyond, and parallel research into the role of ceramics and other vessels can illuminate trends in subsistence practices. New biomolecular data will be critical in identifying diverse culinary traditions of specific social or political spheres within hierarchical societies on the steppe and beyond.

## STAR★Methods

### Key resources table

**Table:** 

REAGENT or RESOURCE	SOURCE	IDENTIFIER
**Software and algorithms**		

Mascot, Matrix Science version 2.7.0.1	Perkins, et al., 1999^63^	matrixscience.com

**Other**		

LC-MS/MS data, including raw, mgf, and mzid files for all samples, positive controls, and extraction blanks are available under accession PXD040519	This paper	https://www.proteomexchange.org/
Curated database of plants, animals, and fermentation bacteria	This paper	Supplementary data

### Resource availability

#### Lead contact

Additional information: Further information and requests for resources should be directed to, Shevan Wilkin (shevan.wilkin@iem.uzh.ch).

#### Materials availability

There are no restrictions on the materials used for this study. All should be available and attainable.

### Method details

#### Methods A: Sample collection

Residue samples were weighed to ∼10 mg and transferred to sterile 2 ml Eppendorf tubes. Each sample was demineralised with 1000 μL EDTA and rotated for 5 days. Following demineralisation, samples were centrifuged at top speed for 10 minutes and 900 μL of the supernatant was removed. The remaining pellet and supernatant were denatured with 6M GuHCl, reduced and alkylated with 10 mM TCEP/CAA, and heated at 99°C for 10 minutes. Magnetic protein binding beads (50:50 mix of Seramag hydrophobic and hydrophilic beads) and 100% ethanol (EtoH) were added to each sample and Thermomixed for 5 minutes at 700 rpm at 24°C. Following this, samples were placed in a magnetic rack and after the protein-bound beads migrated to the magnetic wall the supernatant was removed from each and stored. Beads were then washed three times with 80% EtoH. After the three washes, EtoH was removed and samples were removed from the rack. 100 μL of ammonium bicarbonate (50 mM) was added to each sample, as well as 0.4 μg of Trypsin. Samples were resuspended and remained on the Thermomixer overnight for ∼18 hours at 37°C. Post-digestion, samples were acidified with 5% TFA and peptides were purified on house made StageTips. Each sample was retained on StageTips and sent to the Functional Genomics Centre Zurich at the University of Zurich in Switzerland.

#### Methods B: LC-MS/MS settings

Mass spectrometry analysis was performed on an Orbitrap Exploris 480 mass spectrometer (Thermo Fisher Scientific) equipped with a Nanospray Flex Ion Source (Thermo Fisher Scientific) and coupled to an M-Class UPLC (Waters). Solvent composition at the two channels was 0.1% formic acid for channel A and 0.1% formic acid, 99.9% acetonitrile for channel B. Column temperature was 50°C. For each sample 2 μL of peptides were loaded on a commercial nanoEase MZ Symmetry C18 Trap Column (100Å, 5 μm, 180 μm x 20 mm, Waters) followed by a nanoEase MZ C18 HSS T3 Column (100Å, 1.8 μm, 75 μm x 250 mm, Waters). The peptides were eluted at a flow rate of 300 nL/min. After a 3 min initial hold at 5% B, a gradient from 5 to 22 % B in 90 min and 5 to 35% B in additional 10 min was applied. The column was cleaned after the run by increasing to 95 % B and holding 95 % B for 10 min prior to re-establishing loading condition for another 10 minutes.

The mass spectrometer was operated in data-dependent mode (DDA) with a maximum cycle time of 3 s, using Xcalibur, with spray voltage set to 2.2 kV, funnel RF level at 40 %, heated capillary temperature at 275°C, and Advanced Peak Determination (APD) on. Full-scan MS spectra (350−1,200 m/z) were acquired at a resolution of 120,000 at 200 m/z after accumulation to a target value of 3,000,000 or for a maximum injection time of 45 ms. Precursors with an intensity above 5,000 were selected for MS/MS. Ions were isolated using a quadrupole mass filter with a 1.2 m/z isolation window and fragmented by higher-energy collisional dissociation (HCD) using a normalised collision energy of 30 %. HCD spectra were acquired at a resolution of 30,000 and maximum injection time was set to Auto. The automatic gain control (AGC) was set to 100,000 ions. Charge state screening was enabled such that singly, unassigned and charge states higher than six were rejected. Precursor masses previously selected for MS/MS measurement were excluded from further selection for 20 s, and the exclusion window was set at 10 ppm. The samples were acquired using internal lock mass calibration on m/z 371.1012 and 445.1200.

#### Methods C: Data analysis

Raw MS/MS peptide and protein data files were converted to Mascot Generic Files (MGF) to be searched by Mascot (Matrix Science version 2.7.0.1). Sample MGFs were searched against a database consisting of Swissprot combined with a custom curated dietary protein database (See Supplementary Data for species list). MS/MS ion searches were conducted with trypsin as the digestive enzyme. Carbamidomethyl of cysteine was selected as the fixed modification, with the deamidation of asparagine and glutamine, and the oxidation of methionine as variable modifications. Peptide mass tolerance was set at 10 ppm with an allowance for one ^13^C isotopic shift, and fragment mass tolerance was at 0.01 Da. We allowed for up to three missed cleavages, and the instrument type was set to “Q-Exactive”.

Resulting peptide identifications were filtered with a custom and freely available R script, MS-MARGE https://bitbucket.org/rwhagan/ms-marge/src/master/,^64^ that retains only proteins with at least two distinct peptide spectral matches, peptide e-values below 0.01, and calculates protein and peptide False Discovery Rates (FDR). We aimed for a protein FDR of under 5% and peptide FDR of under 2% for each individual sample, and the actual protein and peptide FDR rates are included in Table S1.

## Data Availability

Data availability: All LC-MS/MS data files (.raw), MGF files (.mgf), and resulting Mascot search files (.dat) have been uploaded to proteomeXchange. Access at https://www.proteomexchange.org/ with accession PXD040519.

## Supplemental information

Document S1. Species and proteomes from the custom curated database created that contains Eurasian plants, animals, and fermentation bacteria proteomes from Uniprot

Table S1. Protein identifications per sample This table lists all dietary peptides recovered per individual, as well as the metadata for each peptide spectral match, the species and tissue for each peptide, and the false discovery rate of each protein dataset.

Table S2. Sample information for each file uploaded to the proteomeXchange
