# Supplementary material for: Curated cauldrons: Preserved proteins from early copper-alloy vessels illuminate feasting practices in the Caucasian steppe
Source: iScience. 2023 Aug 24;26(9):107482. doi: 10.1016/j.isci.2023.107482 (PMC10517358; doi:10.1016/j.isci.2023.107482)
Supplement: Document S1. Species and proteomes from the custom curated database created that contains Eurasian plants, animals, and fermentation bacteria proteomes from Uniprot [file mmc1.pdf]

iScience, Volume *26*

Supplemental information

Curated cauldrons: Preserved proteins from early copper-alloy vessels illuminate feasting practices in the Caucasian steppe

Shevan Wilkin, Peter Hommel, Alicia Ventresca Miller, Nicole Boivin, Antonella Pedergnana, Natalia Shishlina, and Viktor Trifonov

Curated cauldrons: Preserved proteins from early copper alloy vessels illuminate feasting practices in the steppe

Supplementary Information

Supplementary Table 1 – Dietary proteins per individual (separate excel table)
Supplementary Table 2 – Sample list with proteomeXchange file information (separate excel file)
Supplementary Data 1 – fasta file of custom database (separate file)
Supplementary Data 2 – Species included in database

Proteome not found in uniprot.org – The links show proteins found in UNiProtKB (can contain the species of interest as well as other sub-species).

Cereals/ Vegetables/ Fruits/ Spices/ Nuts

Cereals	Common name	Link Uniprot
Hordeum vulgare	Barley	https://www.uniprot.org/proteomes/UP000011116
Avena sativa	Oat	https://www.uniprot.org/uniprot/?query=avena+sativa&sort=score
Secale cereale	Rye	https://www.uniprot.org/uniprot/?query=secale&sort=score
Triticum aestivum	Wheat	https://www.uniprot.org/proteomes/UP000280104
Fagopyrum esculentum	Buckwheat	https://www.uniprot.org/uniprot/?query=buckwheat&sort=score
Setaria italica	Foxtail Millet	https://www.uniprot.org/proteomes/UP000004995
Panicum miliaceum	Proso Millet/ Broomcorn Millet	https://www.uniprot.org/proteomes/UP000275267
Triticum monococcum	Einkorn	https://www.uniprot.org/uniprot/?query=triticum%20monococcum&sort=score
Triticum spelta	Spelt Dinkel wheat	https://www.uniprot.org/uniprot/?query=spelta&sort=score
Triticum turgidum	Emmer Hulled wheat	https://www.uniprot.org/proteomes/UP000324705

Oryza sativa	Rice	https://www.uniprot.org/proteomes/UP000007015
Sorghum bicolor	Sorghum	https://www.uniprot.org/proteomes/UP000000768
Zea mays	Maize (Contamination)	https://www.uniprot.org/proteomes/UP000007305
Amaranthus retroflexus	Redrrot amaranth	https://www.uniprot.org/uniprot/?query=Amaranthus%20retroflexus&sort=score

**Vegetables/ Fruits**	**Common name**	**Link Uniprot**
Vicia faba	Fava bean	https://www.uniprot.org/uniprot/?query=vicia%20faba%20bean&sort=sc
Pisum sativum	Green Peas	https://www.uniprot.org/uniprot/?query=pisum%20sativum&sort=score
Cicer arietinum	Chickpeas	https://www.uniprot.org/proteomes/UP000087171
Lens culinaris	Lentils	https://www.uniprot.org/uniprot/?query=Lens%20culinaris&sort=score
Vicia ervilia	Bitter Vetch	https://www.uniprot.org/uniprot/?query=Vicia%20ervilia&sort=score
Linum usitatissimum	Linseed	https://www.uniprot.org/uniprot/?query=Linum%20usitatissimum&sort=s
Gossypium herbaceum	Cotton Seed	https://www.uniprot.org/uniprot/?query=Gossypium%20herbaceum&sor
Beta vulgaris	Swiss chard	https://www.uniprot.org/proteomes/UP000035740
Allium cepa	Onion	https://www.uniprot.org/proteomes/UP000242203<br><br>https://www.uniprot.org/proteomes/UP000242751
Allium sativum	Garlic	https://www.uniprot.org/uniprot/?query=allium%20sativum&sort=score
Daucus carota	Carrot	https://www.uniprot.org/proteomes/UP000077755
Brassica spp. (*B. oleracea* and others)	Cabbage	https://www.uniprot.org/uniprot/?query=brassica+oleracea&sort=score
Spinacia oleracea	Spinach	https://www.uniprot.org/proteomes/UP000054095
Citrus lemon	Lemons	https://www.uniprot.org/uniprot/?query=citrus%20lemon&sort=score
Citrus sinensis	Sweet orange	https://www.uniprot.org/proteomes/UP000027120
Malus spp.	Apples	https://www.uniprot.org/proteomes/UP000290289

Rubus idaeus	Raspberries	https://www.uniprot.org/uniprot/?query=Rubus+idaeus&sort=score
Pyrus spp.	Pears	https://www.uniprot.org/proteomes/UP000327157
Punica granatum	Pomegranate	https://www.uniprot.org/proteomes/?query=punica&sort=score<br><br>three proteomes
Rheum rhabarbarum	Rhubarb	https://www.uniprot.org/uniprot/?query=rheum+rhabarbarum&sort=score
Phoenix dactylifera	Date	https://www.uniprot.org/proteomes/UP000228380
Prunus spp.	Wild Cherry	https://www.uniprot.org/proteomes/UP000515124
Fragaria viridis	Wild straberry	https://www.uniprot.org/uniprot/?query=Fragaria%20viridis&sort=score
Vaccinium myrtillus	Blueberry	https://www.uniprot.org/uniprot/?query=Vaccinium+myrtillus&sort=score
Ribes nigrum	Black currant	https://www.uniprot.org/uniprot/?query=ribes%20nigrum&sort=score
Ribes rubrum		https://www.uniprot.org/uniprot/?query=ribes%20rubrum&sort=score

**Spices**	**Common name**	**Link Uniprot**
Coriandrum sativum	Coriander	https://www.uniprot.org/uniprot/?query=Coriandrum%20sativum&sort=scor
Brassica nigra	Black mustard	https://www.uniprot.org/uniprot/?query=brassica%20nigra&sort=score
Brassica juncea	Indian mustard	https://www.uniprot.org/uniprot/?query=brassica+juncea&sort=score
Carum carvi	Caraway Persian cumin	https://www.uniprot.org/uniprot/?query=Carum%20carvi&sort=score
Petroselinum crispum	Parsley	https://www.uniprot.org/uniprot/?query=Petroselinum%20crispum&sort=sc
Foeniculum vulgare	Fennel	https://www.uniprot.org/uniprot/?query=fennel&sort=score
Curcuma longa	Turmeric	https://www.uniprot.org/uniprot/?query=curcuma%20longa&sort=score
Cinnamomum verum	Cinnamon	https://www.uniprot.org/uniprot/?query=cinnamomum%20verum&sort=scor
Piper nigrum	Black Pepper	https://www.uniprot.org/uniprot/?query=piper%20nigrum&sort=score
Syzygium aromaticum	Clove	https://www.uniprot.org/uniprot/?query=Syzygium%20aromaticum.&sort=s
Salvia officinalis	Sage	https://www.uniprot.org/uniprot/?query=salvia%20officinalis&sort=score
Mentha ssp.	Mint	https://www.uniprot.org/uniprot/?query=mentha%20piperita&sort=score
Anethum graveolens	Dill	https://www.uniprot.org/uniprot/?query=Anethum%20graveolens&sort=sco

Zingiber officinale	Ginger	https://www.uniprot.org/uniprot/?query=zingiber+officinale&sort=score
Malva spp.	Mallow	https://www.uniprot.org/uniprot/?query=malva&sort=score
Sesamum indicum	Sesame	https://www.uniprot.org/proteomes/UP000504604
Capparis spinosa	Capers	https://www.uniprot.org/uniprot/?query=Capparis&sort=score
Saccharum spp.	Sugar	https://www.uniprot.org/uniprot/?query=saccharum&sort=score
Apis mellifera	Honeybee	https://www.uniprot.org/proteomes/UP000005203
Origanum vulgare	Wild marjoram	https://www.uniprot.org/uniprot/?query=origanum%20vulgare&sort=score
Rosmarinus officinalis	Rosemary	https://www.uniprot.org/uniprot/?query=Rosmarinus+&sort=score

**Nuts**	**Common name**	**Link Uniprot**
Prunus dulcis	Almonds	https://www.uniprot.org/proteomes/UP000327085
Juglands regia	Nuts	https://www.uniprot.org/proteomes/UP000235220
Castanea sativa	Chestnuts	https://www.uniprot.org/uniprot/?query=castanea%20sativa&sort=score
Juglans regia	English Walnut	https://www.uniprot.org/proteomes/?query=Juglans+regia&sort=score
Pistacia vera	Pistachio	https://www.uniprot.org/uniprot/?query=pistacia%20vera&sort=score
Corylus avellana	Hazelnut	https://www.uniprot.org/uniprot/?query=corylus%20avellana&sort=score

**Wild species/ Medicinal species**	**Common name**	**Link Uniprot**
Artemisia vulgaris	Common mugwort	https://www.uniprot.org/uniprot/?query=Artemisia%20vulgaris&sort=sc
Allium ursinum L.	Bear garlic	https://www.uniprot.org/uniprot/?query=Allium%20ursinum%20L.&sor
Allium schoenoprasum	Chives	https://www.uniprot.org/uniprot/?query=Allium%20schoenoprasum&sc
Angelica sylvestris	Wild angelica	https://www.uniprot.org/uniprot/?query=Angelica%20sylvestris&sort=s
Aruncus dioicus	Goat's beard	https://www.uniprot.org/uniprot/?query=aruncus&sort=score

Ornithogalum pyrenaicum	Wild asparagus	https://www.uniprot.org/uniprot/?query=ornithogalum+pyrenaicum&so
Gentiana lutea	Yellow gentian	https://www.uniprot.org/uniprot/?query=Gentiana+lutea&sort=score
Juniperus communis	Common juniper	https://www.uniprot.org/uniprot/?query=Juniperus+communis&sort=sc
Arctium lappa	Greater burdock	https://www.uniprot.org/uniprot/?query=Arctium+lappa&sort=score
Lavandula angustifolia	Lavender	https://www.uniprot.org/uniprot/?query=lavandula+angustifolia&sort=s
Mentha piperita	Peppermint	https://www.uniprot.org/uniprot/?query=mentha+piperita&sort=score
Borago officinalis	Borage	https://www.uniprot.org/uniprot/?query=Borago+officinalis&sort=score
Silybum marianum	Blessed milk-thistle	https://www.uniprot.org/uniprot/?query=Silybum+marianum&sort=scor
Calendula officinalis	Pot marigold	https://www.uniprot.org/uniprot/?query=Calendula+officinalis&sort=sc
Armoracia rusticana	Horseradish	https://www.uniprot.org/uniprot/?query=Armoracia+rusticana&sort=sc
Equisetum arvense	Common horsetail	https://www.uniprot.org/uniprot/?query=Equisetum+arvense&sort=sco
Nasturtium officinale	Watercress	https://www.uniprot.org/uniprot/?query=nasturtium+officinale&sort=sc
Ruta graveolens	Common rue	https://www.uniprot.org/uniprot/?query=Ruta+graveolens&sort=score
Silene vulgaris	Bladder campion	https://www.uniprot.org/uniprot/?query=silene+vulgaris&sort=score
Satureja hortensis	Summer savory	https://www.uniprot.org/uniprot/?query=satureja+hortensis&sort=score
Barbarea vulgaris	Yellow rocket	https://www.uniprot.org/uniprot/?query=Barbarea+vulgaris&sort=score
Veronica allionii	Alpine speedwell	https://www.uniprot.org/uniprot/?query=Veronica+allionii&sort=score
Thymus serpyllum	Thyme	https://www.uniprot.org/uniprot/?query=Thymus+serpyllum&sort=scor
Crataegus monogyna	Hawthorn	https://www.uniprot.org/uniprot/?query=Crataegus+monogyna&sort=s
Cydonia oblonga	Quince	https://www.uniprot.org/uniprot/?query=cydonia+oblonga&sort=score
Lactuca serriola	Prickly lettuce	https://www.uniprot.org/uniprot/?query=Lactuca+serriola&sort=score
Humulus lupulus	European hop	https://www.uniprot.org/uniprot/?query=Humulus+lupulus&sort=score
Malva neglecta	Common mallow	https://www.uniprot.org/uniprot/?query=Malva+neglecta&sort=score
Malva sylvestris	Mallow	https://www.uniprot.org/uniprot/?query=malva+sylvestris&sort=score

Melissa officinalis	Lemon balm	https://www.uniprot.org/uniprot/?query=Melissa+officinalis&sort=score
Urtica dioica	Great nettle	https://www.uniprot.org/uniprot/?query=urtica+dioica&sort=score
Rubus caesius	European dewberry	https://www.uniprot.org/uniprot/?query=Rubus+caesius&sort=score
Papaver rhoeas	Common poppy	https://www.uniprot.org/uniprot/?query=Papaver+rhoeas&sort=score
Pastinaca sativa	Wild parsnip	https://www.uniprot.org/uniprot/?query=Pastinaca+sativa&sort=score
Plantago major	Common plantain	https://www.uniprot.org/uniprot/?query=plantago+major&sort=score
Lathyrus sylvestris	Flat pea	https://www.uniprot.org/uniprot/?query=lathyrus+sylvestris&sort=score
Pulmonaria officinalis	Lungwort	https://www.uniprot.org/uniprot/?query=pulmonaria+officinalis&sort=s
Cichorium intybus	Chicory	https://www.uniprot.org/uniprot/?query=Cichorium+intybus&sort=score
Rosa canina	Dog rose	https://www.uniprot.org/uniprot/?query=rosa+canina&sort=score
Sambucus nigra	European elder	https://www.uniprot.org/uniprot/?query=Sambucus+nigra&sort=score
Chenopodium bonus henricus	Good King Henry Poor-men asparagus	https://www.uniprot.org/uniprot/?query=Chenopodium+bonus+henricu
Lapsana communis	Common nipplewort	https://www.uniprot.org/uniprot/?query=Lapsana+communis&sort=sco
Taraxacum officinale	Common dandelion	https://www.uniprot.org/uniprot/?query=Taraxacum+officinale&sort=s
Trifolium pratense	Red clover	https://www.uniprot.org/uniprot/?query=Trifolium+pratense&sort=scor
Helianthus tuberosos	Topinambour	https://www.uniprot.org/uniprot/?query=Helianthus+tuberosus&sort=s
Valerianella locusta	Corn salad	https://www.uniprot.org/uniprot/?query=Valerianella+locusta&sort=sco
Viola odorata	Sweet violet	https://www.uniprot.org/uniprot/?query=Viola+odorata&sort=score
Laurus nobilis	Laurel	https://www.uniprot.org/uniprot/?query=laurus+nobilis&sort=score
Artemisia absinthium		https://www.uniprot.org/uniprot/?query=artemisia+absinthium&sort=s
Achillea millefolium	Yarrow	https://www.uniprot.org/uniprot/?query=Achillea&sort=score

Fish/Egg

Clupea herengus	Atlantic Herring	https://www.uniprot.org/proteomes/UP000515152
Gadus morhua	Atlantic Cod (Stockfish)	https://www.uniprot.org/proteomes/UP000305155
Esox lucius	Pike	https://www.uniprot.org/proteomes/UP000265140
Cyprinus carpio	Common Carp	https://www.uniprot.org/proteomes/?query=Cyprinus+carpio+&sort=score
Abramis brama	Bream	https://www.uniprot.org/uniprot/?query=abramis%20brama&sort=score
Perca fluviatilis	European Perch	https://www.uniprot.org/proteomes/UP000465112
Salmo trutta	Brown Trout	https://www.uniprot.org/proteomes/UP000472277
S. marmoratus	Marble trout	https://www.uniprot.org/uniprot/?query=salmo%20marmoratus&sort=score
Gallus gallus	Chicken-Eggs	https://www.uniprot.org/proteomes/UP000000539

Organisms involved in the Fermentation of Foodstuff and Intoxication

Bacteria or Fungi	Common name/function/ disease	Link Uniprot
Claviceps purpurea fungus	Ergot fungus (ergotism)	https://www.uniprot.org/proteomes/UP000016801
Saccharomyces cerevisiae	Yeast (winemaking, backing, brewing)	https://www.uniprot.org/proteomes/UP000002311
Lactobacillus brevis	Plant fermentation	https://www.uniprot.org/proteomes/?query=Lactobacillus+brevis&sort=score
Lactobacillus bulgaricus	Plant fermentation	https://www.uniprot.org/proteomes/?query=Lactobacillus+bulgaricus&sort=score
Lactobacillus lactis	Plant fermentation	https://www.uniprot.org/proteomes/?query=Lactobacillus+lactis&sort=score

Streptoc. thermophilus	Plant fermentation	https://www.uniprot.org/proteomes/UP000004150
Lactobacillus delbrueckii	Plant fermentation	https://www.uniprot.org/proteomes/?query=Lactobacillus+delbrueckii&sort=score
Lactobacillus salivarius	Plant fermentation	https://www.uniprot.org/proteomes/?query=Lactobacillus+salivarius&sort=score
Lactobacillus acidophilus	Plant fermentation	https://www.uniprot.org/proteomes/?query=Lactobacillus+acidophilus&sort=score
Lactobacillus casei	Plant fermentation	https://www.uniprot.org/proteomes/?query=lactobacillus+casei&sort=score
Lactobacillus curvatus	Plant fermentation	https://www.uniprot.org/proteomes/?query=Lactobacillus+curvatus&sort=score
Lactobacillus buchneri	Plant fermentation	https://www.uniprot.org/proteomes/?query=Lactobacillus+buchneri&sort=score
Lactobacillus confusus	Plant fermentation	https://www.uniprot.org/proteomes/?query=Lactobacillus+confusus&sort=score
Lactobacillus fermentum	Plant fermentation	https://www.uniprot.org/proteomes/?query=lactobacillus+fermentum&sort=score
Lactobacillus sakei	Plant fermentation	https://www.uniprot.org/uniprot/?query=Lactobacillus%20sake&sort=score
Lactobacillus plantarum	Plant fermentation	https://www.uniprot.org/uniprot/?query=Lactobacillus+plantarum&sort=score
Lactobacillus pentosus	Fermentation	https://www.uniprot.org/proteomes/?query=Lactobacillus+pentosus&sort=score
Leuconostoc paramesenteroides	Plant fermentation	https://www.uniprot.org/proteomes/?query=Leuconostoc+paramesenteroides&sort=score
Enterococcus faecium	Plant fermentation	https://www.uniprot.org/proteomes/?query=Enterococcus+faecium&sort=score
Enterococcus faecalis	Plant fermentation	https://www.uniprot.org/proteomes/?query=Enterococcus+faecalis&sort=score
Streptococcus bovis	Plant fermentation	https://www.uniprot.org/proteomes/?query=Streptococcus+bovis&sort=score

Pediococcus acidilactici	Plant fermentation	https://www.uniprot.org/proteomes/?query=Pediococcus+acidilactici&sort=score
Pediococcus pentosaceus	Plant fermentation	https://www.uniprot.org/proteomes/?query=Pediococcus+pentosaceus&sort=score
Leuconostoc mesenteroides subsp. dextranicum	Fermentation dairy products<br><br>Fermentation (sauerkraut and pickles)	https://www.uniprot.org/proteomes/?query=leuconostoc+dextranicum&sort=score
Streptococcus lactis	Fermentation dairy products	https://www.uniprot.org/proteomes/?query=Streptococcus+lactis&sort=score
Brevibacterium	Fermentation dairy products	https://www.uniprot.org/proteomes/?query=Brevibacterium&sort=score
Lactobacillus delbrueckii bulgaricus	milk	https://www.uniprot.org/proteomes/?query=Lactobacillus+delbrueckii+bulgaricus&sort=score
Streptococcus salivarius	milk	https://www.uniprot.org/proteomes/?query=streptococcus+salivarius&sort=score
